# An emerging novel goose astrovirus associated with gosling gout disease, China

**DOI:** 10.1038/s41426-018-0153-7

**Published:** 2018-09-05

**Authors:** Xinyu Zhang, Dan Ren, Tuofan Li, Huayan Zhou, Xiaoyu Liu, Xiaobo Wang, Hao Lu, Wei Gao, Yajuan Wang, Xiaoyan Zou, Huaichang Sun, Jianqiang Ye

**Affiliations:** 1 College of Veterinary Medicine, Yangzhou University, Yangzhou, 225009 Jiangsu China; 2 Jiangsu Co-innovation Center for Prevention and Control of Important Animal Infectious Diseases and Zoonoses, 225009 Yangzhou, Jiangsu China; 3 Joint International Research Laboratory of Agriculture and Agri-Product Safety, the Ministry of Education of China, Yangzhou University, 225009 Yangzhou, Jiangsu China; 4 Institutes of Agricultural Science and Technology Development, Yangzhou University, 225009 Yangzhou, Jiangsu China

## Abstract

Since the first isolation from human, astroviruses have been detected in many species. Wide host range and occasional cross-transmission of astrovirus pose a risk for zoonotic infection. Here, novel astroviruses were identified from goslings with recent epidemic gout disease in China. A virus, designated as GD, was efficiently isolated from a diseased gosling using LMH cells. Genome of GD amplified using 5′ and 3′ RACE was 7183nt in full length. Sequence analysis revealed the genome of GD was <60.8% homology with others deposited in Genbank. Moreover, GD could be neutralized by goose convalescent sera, and the gout associated symptom in goslings could be reproduced by GD infection. Our data demonstrated the goose astrovirus could be one of the causative agents of the ongoing gosling gout disease in China. The identification of the goose astrovirus not only diversified the astrovirus species, but also broadened the disease patterns caused by astroviruses.

## Introduction

Astroviruses are currently classified into two genera, Mamastroviruses (MAstVs) and Avastroviruses (AAstVs)^1^. MAstVs mainly infect mammals including human, ovine, bovine, porcine, feline, canine, mink, bat, deer, mouse, sea lion, dolphin etc., whereas AAstVs generally infect aviansuch as turkey, chicken, duck, pigeon, and goose^1,2^. Notably, genetic variation and cross-species transmission of astrovirusespose the risk for zoonotic infection^2,3^. Infection with astroviruses mainly cause enteric diseases such as gastroenteritis and diarrhea in human and animals as reported initially, then nephritis in chicken and pigeon, hepatitis in ducklings, and encephalitis in human, cattle, and sheep recently, which broadens the disease pattern of astroviruses and highlights its significance^2,4–6^. In 2015, a gout disease emerged in 1-week-old goslings in Anhui province, which had spread to most provinces of China by 2017 with high morbidity (80–90%) and mortality (20–70%), and no pathogenic bacteria could be isolated from the diseased goslings. However, little is known about the pathogen for the goose gout disease endemic in goose flocks in China. During 2011–2012, the outbreaks of gout disease were reported in broilers in India. Through virus isolation and infection study, Bulbule et al. demonstrated that a novel chicken astrovirus (CAstV) could be as one of the causative agents for the gout disease in chicken flocks in India^7^. To control the spread of the goose gout disease, we investigated the pathogenic agent of the disease. Through in vitro and in vivo experiments, we identified and isolated a novel goose astrovirus different from the CAstVas a causative agent of the gout disease recently circulating in gosling flocks in China.

## Results

### Outbreaks of gout disease in goslings in China since 2015

**Fig. 1:**
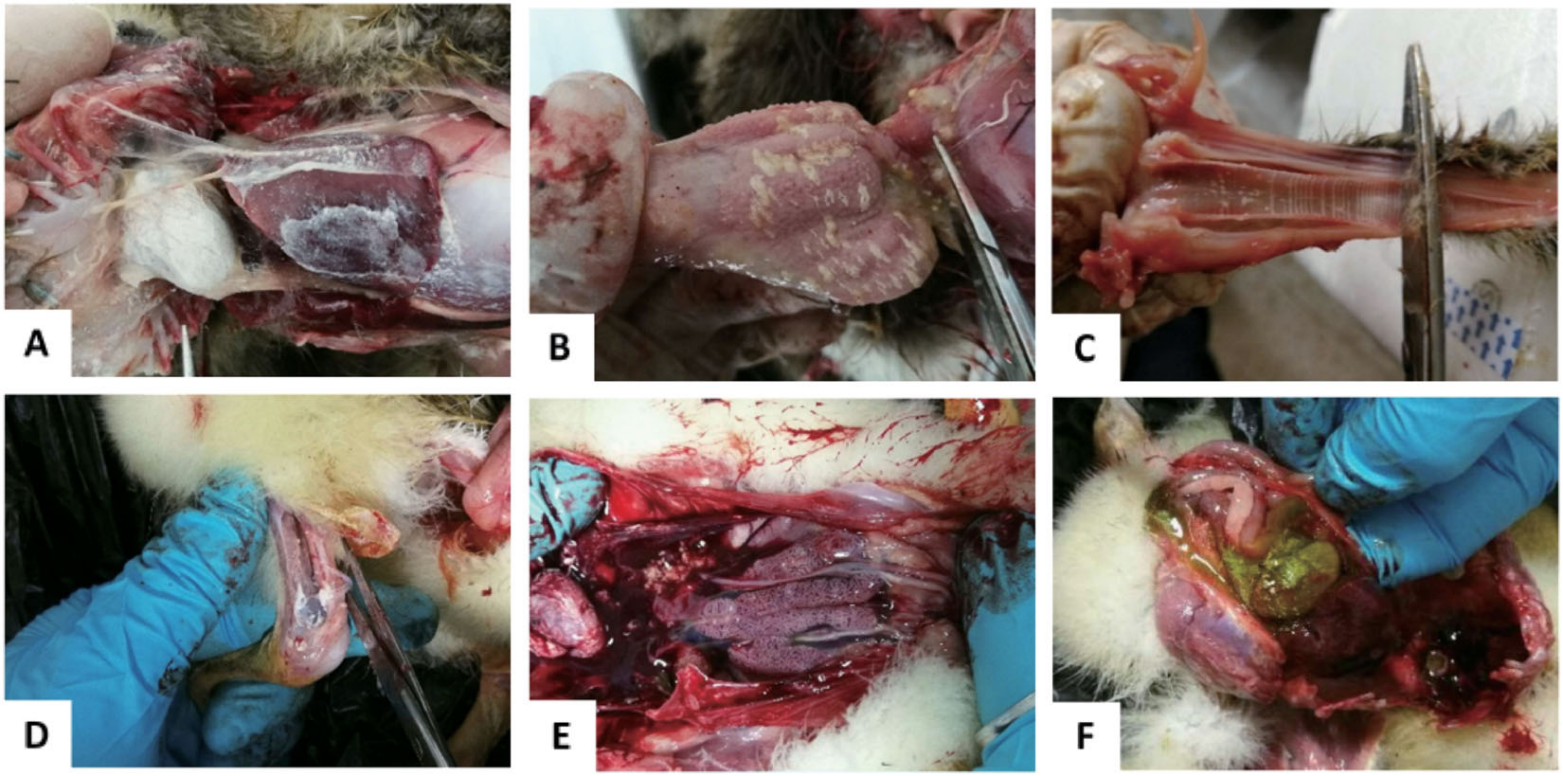
Gross pathologic changes in diseased goslings. **a** Deposit of urate on the surface of heart and liver. **b** Deposit of urate on the glandular gastric mucosa. **c** Deposit of urate on the trachea mucous membrane. **d** Deposit of urate in the leg joint. **e** Renal enlargement. **f** Deposit of urate in the gallbladder

In 2015, a gout disease emerged in 1-week-old goslings in Anhui province, which has spread to most provinces of China by 2017. The outbreak of the gosling gout disease has caused significant economic loss in goose industry. The clinical signs of the disease were characterized by white feces, leg joint enlargement with urate deposits and paralysis. At necropsy, kidney enlargement and intensive urate deposits were found in the gallbladder, knees, and ureters, and on the surfaces of cardiac, heart, liver, air sacs, trachea, and proventriculus (Fig. 1a–f). The disease lasted for 7–10 days with high morbidity and mortality. The survival goslings grew slowly and were susceptible to bacterial infection.

### Discovery of novel astroviruses from the goslings with gout disease

To investigate the causative agent for the ongoing gosling gout in China, 49 diseased goslings with gout symptom were collected from 10 goose flocks from Jiangsu, Anhui, Shandong, Guangdong, and Fujian Provinces. PCR/RT-PCR was performed using specific primers listed in Table 1 to detect viral nucleic acids. Except for a 434 bp band amplified from all samples (*n* = 30) by a pair of degenerate primers to ORF1b gene of avian astrovirus, negative results for the other viruses were demonstrated. However, the samples (*n* = 5) detected from the healthy goslings without gout disease, provided by National water fowl gene bank, Taizhou, China, were negative by RT-PCR in avian astrovirus detection. The 10 amplicons from the 10 flocks respectively designated as AHLA, AHDY, FJZA, GDHZ, JSXZ, JSGY, JSDY, JSYZ, SDJM, and SDLJ, were sequenced directly for homology analysis. Results indicated the 10 sequences of the PCR products were highly consistent (å 99%), but had <70% homology with those deposited in GenBank. Furthermore, the sequences detected shared only 60.2–62% homology with the astrovirus FLX (NC_034567) recently identified in goose with enteritis from China^8^.

**Table 1:** Primers used in this study for detection of the viruses

Primer name	Sequence (5′–3′)	Target	Amplicon size	Reference
FAdV F	CAACTACATCGGGTTCAGGGATAACTTC	Hexon gene of fowl adenovirus	766bp	^24^
FAdV R	CCAGTTTCTGTGGTGGTTGAAGGGGTT			
GHPV F	GAGGTTGTTGGAGTGACCACAATG	VP1 gene of goose hemorrhagic polyomavirus	144bp	^25^
GHPV R	ACAACCCTGCAATTCCAAGGGTTC			
GRV F	TGAGACGCCTGACTACGATT	Sigma C gene of goose reovirus	380bp	^26^
GRV R	ATGCTTGGAGTGAGACGACT			
GCoV F1	TATATCTGCAAAGAATAGGGCTCG	ORF1b gene of goose coronavirus	208bp	^27^
GCoV R1	GCCCATAAGCATAGGATCGTCAACG			
GCoV F2	TGGCTCCAGGAAAAGATGTTGA	NC gene of goose coronavirus	495bp	^27^
GCoV R2	CTCCCCAATTCACTCTATCTG			
DTMUV F	GCCACGGAATTAGCGGTTGT	E gene of duck Tembusu virus	401bp	^28^
DTMUV R	TAATCCTCCATCTCAGCGGTGTAG			
GPVF	AGACTTATCAACAACCATCAT(C) T	VP1 gene of goose parvovirus	779bp	^29^
GPVR	TCACTTATTCCTGCTGTAG			
AvAstV F	GAYTGGACNMGNTAYGAYGGNACNATNCC	ORF1b gene of avian astrovirus	434bp	^30^
AvAstV R	YTTNACCCACATNCCRAA			
GSP1	gattacgccaagcttCATATCTATGACGACATTCGTGTC	Gene specific primer targeting to RdRp for 5′-RACE	/	This study
NGSP1	gattacgccaagcttCCTGTCCAACCATCATAAGACACC			
GSP2	gattacgccaagcttAAGCCTCTCTTCTGGCGGATAC	Gene specific primer targeting to RdRp for 3′-RACE	/	This study
NGSP2	gattacgccaagcttGACACAAGCCTATCATCGCCATAG			

### Whole genome analysis of the novel goose astrovirus

**Fig. 2:**
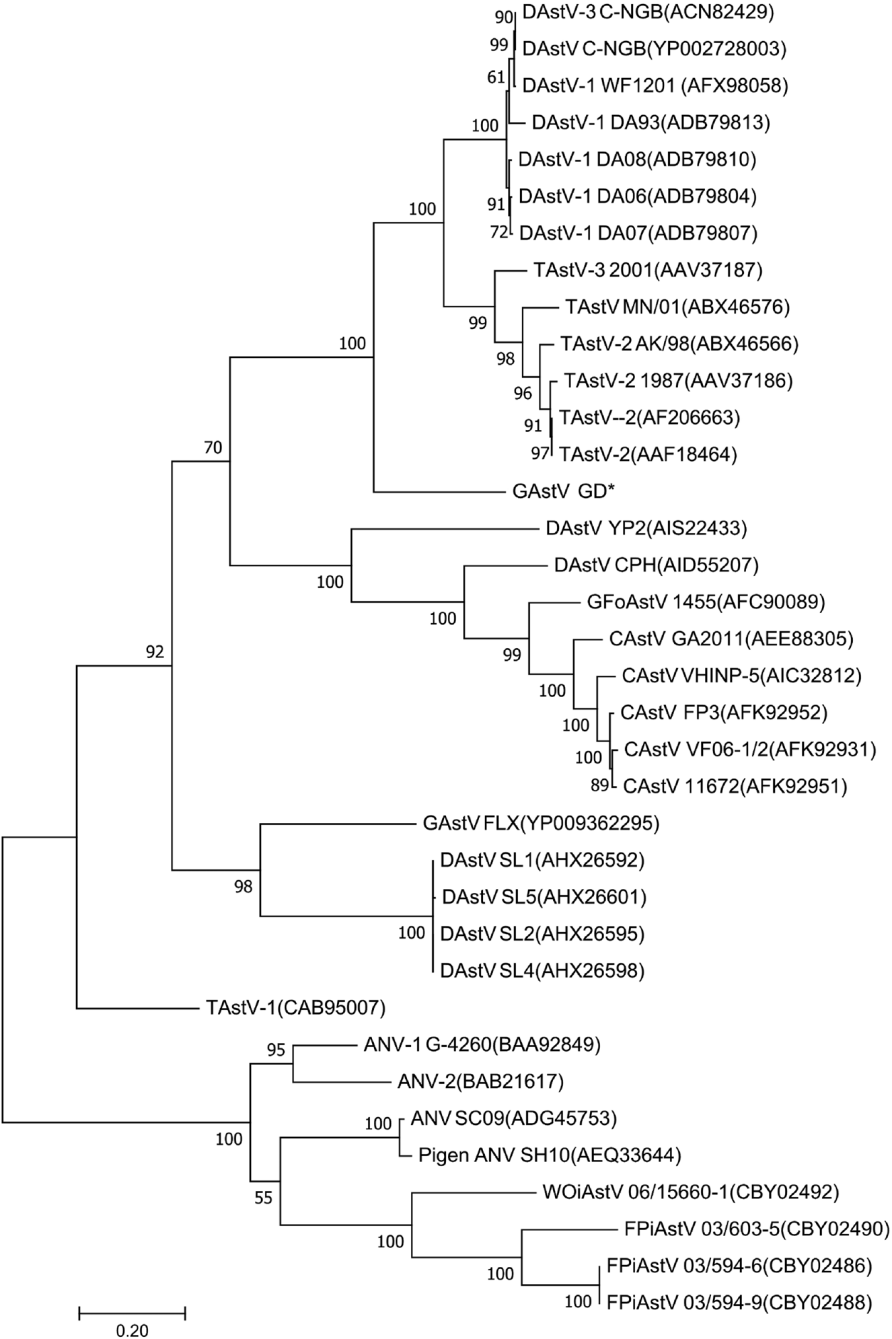
Phylogenetic tree analysis by MEGA7. Amino acid sequence encoded by ORF2 of avastrovirus GD labeled with star

To further determine the molecular characteristics of the goose astrovirus, the viral RNA genome was amplified by RACE-PCR from the GDHZ sample. Sequencing data showed that the viral RNA genome was 7183 nt in length (GenBank accession no. MG934571) with a typical genome structure of astroviruses^8,9^. Between the 18-nt 5′ untranslated region (UTR) and 236-nt 3′ UTR, three open reading frames (ORFs) were predicted, including 3225-nt ORF1a, 1287-nt ORF1b, and 2115-nt ORF2. A potential ribosomal frameshift signal (AAAAAAC) was identified downstream of ORF1a, followed by a UAG stop codon. As expected, the conserved CCGAA motif of astroviruses was found between ORF1b and ORF2. Sequence alignment of the RNA genome showed <60.8% homology with other AAstVs. To gain further insight into the evolutionary relationship of the virus with other AAstVs, phylogenetic tree was constructed based on the sequences of ORF2. The novel astrovirus formed a distinct clade in *Avastroviruses 3* including TAstV-2 (AAF18464), TAstV-2 AK/98 (ABX46566), and DAstV-3 C-NGB (ACN82429) as described in Fig. 2. Recombinant analysis was conducted among all the genomes of astroviruses deposited in Genbank, and the results demonstrated no recombinant event happened in the viral genomic sequence.

### Efficient isolation of the novel goose astrovirus in LMH cells

**Fig. 3:**
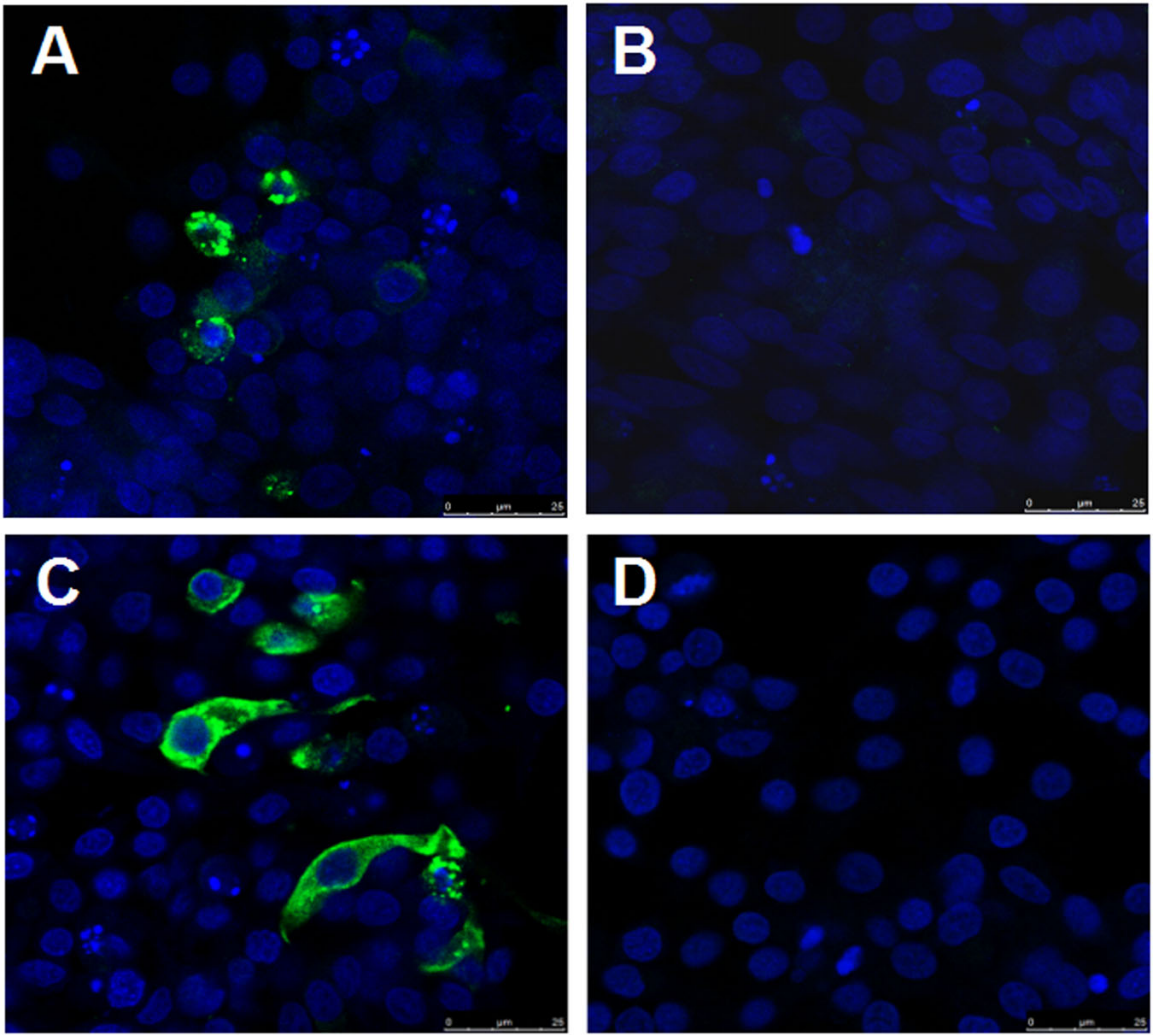
Identification of the novel goose astrovirus GD isolate in LMH cells by indirect immunofluorescence assay. **a**, **b** LMH cells infected with or without GD were reacted with the convalescent sera from the survival geese with gout symptomrespectively. **c**, **d** LMH cells infected with or without GD were reacted with the mouse sera against capsid P2 of GD respectively

To isolate the novel goose astrovirus, the homogenates of the pooled liver and kidney from the diseased gosling were inoculated into chicken liver cell line LMH. After five passages, an astrovirus was isolated and identified by RT-PCR using the above primers, named as GD. To further confirm the isolation of the virus, the infected LMH cells were analyzed through indirect fluorescent assay (IFA) using the convalescent sera from the survival geese with gout symptom and the mouse sera against the capsid P2 of GD (generated in our laboratory) respectively. As described in Fig. 3, the specific fluorescent signals could be found in the cytoplasm of the LMH cells infected with GD, but not in the control LMH cells. Notably, there was no cytopathic effect found in infected LMH cells. Moreover, GD virus could be efficiently neutralized by the convalescent sera from the survival goose with gout disease, and the neutralization titer of the goose convalescent sera against GD reached 1:3200, however, the sera from healthy geese did not show any neutralization activity, which highlighted the association between the novel goose astrovirus and the gosling gout disease. All these clearly demonstrate that a novel goose astrovirus GD has been efficiently isolated from the diseased goslings with severe gout symptoms.

### Infection study with the novel goose astrovirus GD

**Fig. 4:**
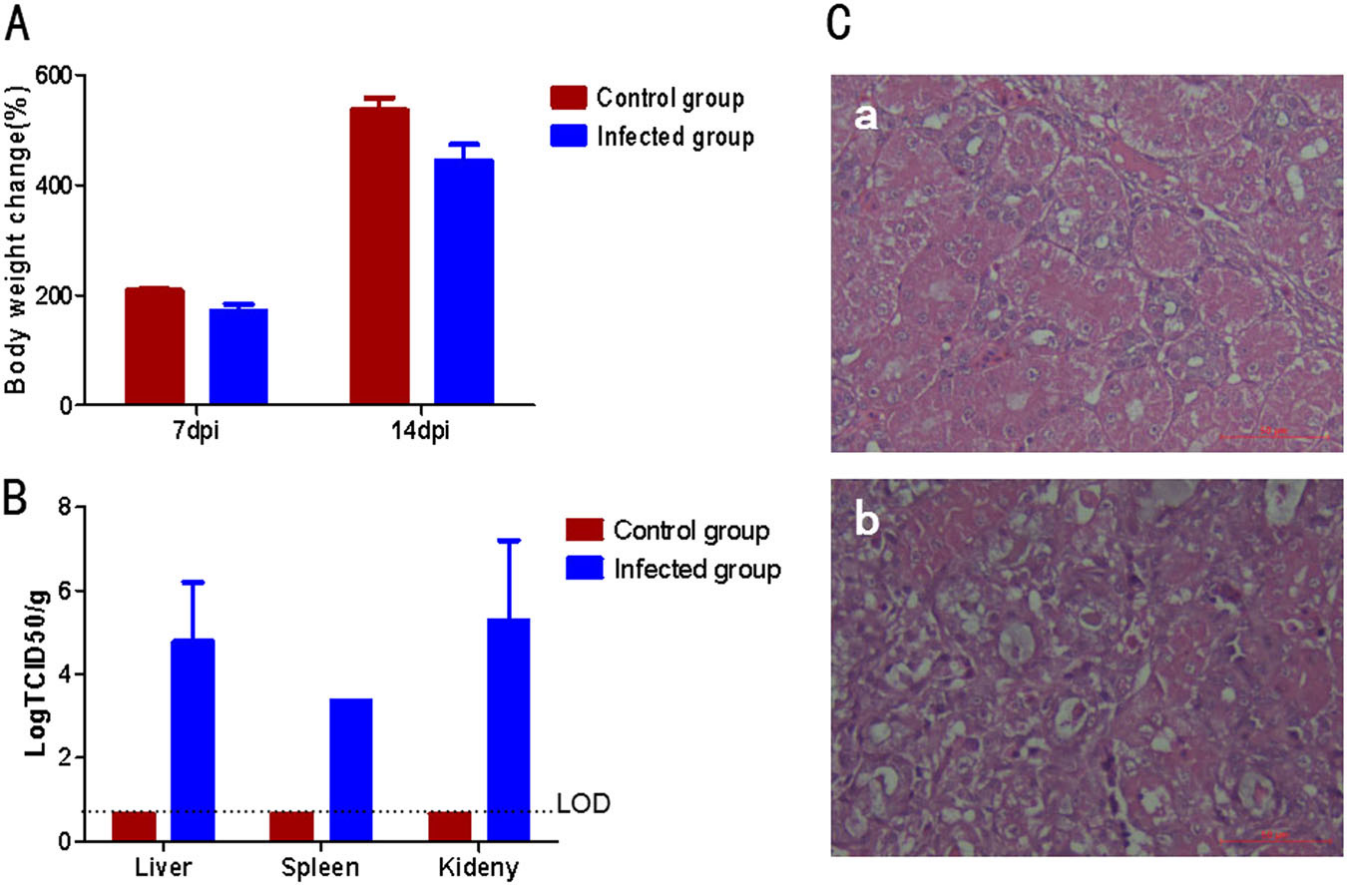
Infection study with the novel goose astrovirus GD. **a** Bodyweight changes of the infected goslings. **b** Viral titer in the tissues from the infected goslings. **c** Histopathologic changes in infected goslings. a Normal kidney from the non-infected gosling, b Kidney from the infected gosling with renal tubular necrosis, abscission of renal tubular epithelial cells and protein cast in renal tubules

To investigate whether the GD isolatewould be the causative agent for the ongoing goslings gout in China, 5-day-old goslings were infected with GD. All the infected goslings showed clinical signs with depression and started to excrete white feces at day 3 post infection, and grew slower than the non-infected control goslings as described in Fig. 4a. At day 7 post infection, three inoculated goslings were killed and the tissues were collected. At necropsy, the typical gout pathological changes were found, including kidney with swelling and white substance-filled ureters. The histopathological assay also demonstrated the similar pathological signs to that of naturally infected goslings, including necrosis and abscission of renal tubular epithelial cells, presence of protein cast in renal tubules (Fig. 4c). Moreover, the virus could be efficiently isolated from the liver, spleen and kidney of the infected goslings at day 7 post infection, but not from the non-infected control goslings as described in Fig. 4b. Although all the infected goslings had clinical signs and histopathological changes associated with gout, all these infected goslings were survival during 2 weeks observation post infection. In addition, the neutralizing antibody titer against GD from the infected goslings could reach 1:6400 at day 14 post infection. All these data demonstrated that the GD isolate could efficiently infect the goslings and cause diseases associated with gosling gout.

## Discussion

It has been reported that avastrovirus infections can cause enteritis, diarrhea, hepatitis of ducklings, nephritis of chickens, and decrease of egg-hatching rate of different fowls^7,10–17^. Based on the amino acid sequences of the ORF2-encoded capsid protein, these AAstVs can be divided into three species: *Avastroviruses 1*, *2*, and *3* (The International Committee on Taxonomy of Viruses, ICTV, 2016). In this study, a novel goose astroviruses GD was identified and isolated from goslings with gout symptom. The infection study further confirmed that the GD belonging to *Avastroviruses 3* could be the causative agent of the ongoing gosling gout disease in China. Although CAstVs could induce gout disease in chickens^7^, CAstVs have only about 35.1–35.8% homology with GD on amino acid sequence of capsid, and there is no recombinant event happened between them, which indicate that the GD is an under recognized novel astrovirus. In comparison with the goose astrovirus recently identified in geese with enteritis in China^8^, the novel goose astrovirus showed only about 60% identity in nucleic acid. This highlights at least two species of goose astrovirus associated with different diseases are circulating in goslings flocks in China.

The infection study with GD could reproduce the clinical signs and histopathogenesis associated with the gout disease, but all the infected goslings were survival during 2 weeks observation post infection. The high morbility and low mortality for the infection study with GD might be related with the infection route and dose, and the age. Although the pathogens including fowl adenovirus, goose hemorrhagic polyomavirus, goose reovirus, goose coronavirus, goose parvovirus, and duck Tembusu virus were not able to be detected in the diseased goslings, we could not exclude the potential co-infections with other pathogens, which may exacerbate the clinical symptoms of gout. In addition, whether the high protein level in the feed promotes the occurrence of gout also need to be further investigated. The high-titer neutralizing antibody to GD from the survival geese highlighted the novel goose astrovirus could efficiently induce enough adapt immunity in goslings. However, whether this adapt immunity can protect goslings completely from the novel goose astrovirus need to be further studied.

The mechanism for the gout disease induced by either CAstV reported by Bulbule et al. or GD identified in this study need to be further elucidated. Moser et al. reported that astroviruses could increase the permeability of the epithelial cells^18^. The increased permeability of the kidney epithelial cells induced by the astroviruses might contribute to the gout disease^7^. In addition, due to lacking arginase in poultry, ammonia cannot be processed into urea instead of purine, hypoxanthine, and xanthine, then oxidized to uric acid, forming sodium urate and calcium urate, excreted through kidney finally. If the rate of urate formation is greater than the excretory capacity of the urinary organs, gout can be caused with the urate deposits on visceral surfaces^19^. Therefore, factors that cause renal and urinary tract injury or urine concentration and urinary excretion disorder can promote the formation of urate deposits. In this study, the novel astrovirus GD could cause kidney damage, which maybe the main cause of gosling gout in China.

In summary, this is the first demonstration of a gout-disease associated novel goose astrovirus efficiently isolated by LMH cells. The specific sequences of the astroviruses detected in samples from the five different provinces demonstrated the widespread of the novel goose astroviruses in China. However most astrovirusesare difficult to grow in cell culture^20^, the novel goose astrovirus GD could be isolated efficiently in LMH cells in this study, which provides an efficient proliferation system for the avastrovirus in vitro. This efficient system might provide a good tool for developing inactivated vaccine and investigating the pathogenicity mechanism of goose astrovirus in the future. The full genomic RNA of the novel goose astrovirus was sequenced and publicated in Genbank,which pave ways for further developing vaccines and diagnostic methodsin controlling the gout disease in goslings. During the preparation of the revised version for this manuscript, other groups in China recently also reported the novel goose astrovirus^21–23^. Different from those recently reported by other groups, we efficiently isolate such novel goose astrovirus in vitro using the cell culture system (LMH cells) and reproduce the associated gout disease by inoculating the cell cultured virus GD. Of course, the originator, host range, variation, molecular pathogenesis, and potential zoonotic infection of the novel goose astrovirus need to be further studied.

## Materials and methods

### Tissue and sera

Fresh tissues (such as liver, kidney, trachea, etc.) were collected from diseased goslings (*n* = 49) aged 10–15 days with gout symptom from 10 different goose flocks in Jiangsu, Anhui, Shandong, Guangdong, and Fujian Provinces during July to November 2017. The convalescent sera were collected from survived geese (*n* = 6) aged 55 days suffered gout disease or survived goslings (*n* = 9) experimentally infected with astrovirus.

### Viral nucleic acids detection

Total DNA and RNA of pooled liver and kidney were extracted using QIAmp DNA Mini Kit (Qiagen) and Trizol reagent (Invitrogen) respectively based on protocols from the manufacturers. PCR/RT-PCR for the detection of fowl adenovirus, goose hemorrhagic polyomavirus, goose reovirus, goose coronavirus, duck Tembusu virus, goose parvovirus, and avian astrovirus were performed using the primers listed in Table 1^24–30^, and PCR/RT-PCR kits from Takara Biotechnology Co., Ltd (Dalian). The amplified PCR fragments were sequenced by Sangon Biotech (Shanghai) Co., Ltd.

### Viral genome sequencing and analysis

To determine the full-length nucleotide sequences of virus, RACE-PCR was performed using RNA extracted from pooled liver and kidney with a SMARTer® RACE 5′/3′ Kit (Takaka Bio USA, Inc). The 5′ and 3′ end fragments were amplified using genome-specific primer GSP1, NGSP1, and GSP2, NGSP2 listed in Table 1. Then PCR products were cloned into the pRACE vector and sequenced according to the manufacturer’s protocol. ORFs prediction and special sequences search in the genome were conducted by Lasergene7. Nucleic acid and amino acid sequences identity analyses was conducted by Protein BLAST in GenBank. Phylogenetic tree was constructed by MEGA7. Recombinant analysis was performed by RDP4.39 and SimPlot3.5.1.

### Virus isolation

The homogenate of the pooled liver and kidney samples from a diseased gosling were filtered with 0.22 μm filter, then 1mL filtrate mixed TPCK (l-1-tosylamido-2-phenylethyl chloromethyl ketone)-treated trypsin at the concentration of 1 μg/mL was inoculated into chicken liver cell line (LMH, ATCC) with 80% confluence in 6 wells plate. 2 h post infection, the supernatant was discarded, and OPTI-MEM medium containing 1 μg/mL TPCK trypsin was added. The infected cells were blindly passaged every 4 days, and the viral antigen was detected by immunofluorescent assay.

### Antiserum preparation

According to the viral genome sequence, a codon-optimized gene of capsid P2 was synthesized and cloned into pET-30a (Novagen). The recombinant capsid P2 protein was expressed in *E.coli* BLR (DE3) cells (Novagen) under the inducing of isopropyl-*β*-d-thiogalactopyranoside (Sangon Biotech) at concentrate of 1mmol/L and purified using Ni-NTA Agarose (Qiagen). After verified using convalescent goose sera by Westernblot, 200 μg purified recombinant protein was inoculated to 8-week old Balb/c mouse in abdomen at an interval of 10 days. 10 days after the 3rd immunization, the mouse blood was collected to prepare antisera.

### Indirect immunofluorescence assay

The infected cells were fixed with 4% paraformaldehyde (Sangon Biotech) for 10 min, followed by rinsing with PBS. 0.2% v/v triton X-100 (Sigma) diluted in PBS was applied to each well for 10 min. After washing three times with sterile PBS, the cells were overlaid with PBS containing 5% w/v bovine serum albumin (Sangon Biotech) and incubated for 30 min at 37° C. The mouse antiserum anti-capsid P2 was used as the primary antibody with a 1:200 dilution in PBS. After 45min incubation at 37 °C, the cell monolayers were rehydrated by rinsing three times with PBS. Coverslips were stained using goat anti-mouse IgG conjugated with FITC (KPL) and incubated for a further 45 min at 37° C. The stained cells were rinsed with PBS, then dyed with Hoechst33342 (Solarbio) and rinsed again. The stained cells were then examined under a confocal microscope.

### Virus neutralization test

The virus neutralization test was performed as reported^31^. Briefly, the convalescent sera both from the clinical survived goslings (*n* = 6) with gout disease and the experimental infected survived goslings (*n* = 9) were serially diluted with OPTI-MEM medium. Then, 50 µL of the diluted sera were mixed with an equal volume of 200 TCID_50_ virus. The mixture was incubated for 1 h at 37° C, then inoculated into LMH cells and the inoculated cells were washed once with PBS. At day 4 post inoculation, the inoculated cells was analyzed by indirect immunofluorescence assay to determine the neutralization titer of the sera.

### Infection study in vivo

To determine pathogenicity of the isolated virus, twelve 5-day-old goslings were inoculated with the novel goose astrovirus (5×10^4^ TCID_50_) through muscle injection routes and twelve non-inoculated 5-day-old goslings were used as control. The clinical signs and the bodyweight of these goslings were observed and monitored after inoculation. Three infected goslings were killed at day 7 post infection for detecting the viral titer in tissues and the histopathological analysis.

### Histopathology

Fresh tissues collected from the diseased or experimental goslings were fixed with 10% neutral buffered formalin. Then paraffin sections were prepared and stained with haematoxylin and eosin (H&E) as described^16^. Histopathological changes in the tissues were observed under an optical microscope.
